# HIGH-INTENSITY INTERVAL TRAINING, SLEEP, AND COGNITION IN OLDER ADULTS: A SYSTEMATIC REVIEW

**DOI:** 10.1093/geroni/igad104.2945

**Published:** 2023-12-21

**Authors:** Carleara Weiss, Rebecca Lorenz

**Affiliations:** University at Buffalo, Buffalo, New York, United States; University at Buffalo, Getzville, New York, United States

## Abstract

Sleep disturbances have been linked to cognitive and physical decline in older adults. High intensity interval training (HIIT) may resynchronize disrupted sleep-wake cycles, improve sleep quality, and potentially delay detrimental impacts of aging on cognition and physical performance. Comprehensive reviews about the impact of HIIT on cognitive and physical decline in older adults are lacking. A systematic review was conducted using the Preferred Reporting Items for Systematic Reviews and Meta-Analyses (PRISMA) approach to evaluate the impact of HIIT upon sleep and cognition. PubMed, Scopus, and Web of Science were searched for articles. Six publications representing four randomized controlled trials met the inclusion criteria. Data from intervention and control groups included 158 participants, predominately female (70.4%) with median age of 69.5 years. Exercise modalities varied across studies; however, duration of 12-weeks was consistent. HIIT reduced objectively measured sleep onset latency and lowered wake after sleep onset compared to no activity and regular physical activity. However, changes in sleep quality with HIIT did not differ from those achieved with stretching exercise and moderate-intensity physical activity. In addition, only one study investigated the impact of HIIT on both sleep and cognition, indicating improvement in cognitive performance and memory but no significant changes in sleep efficiency. Despite the potential for HIIT to improve sleep outcomes, memory, and cognition, more studies are warranted, particularly those that include diverse populations and larger sample sizes, prior to forming recommendations for use in clinical settings and communities.

